# Spinal Epidural Abscess Complicated by Compressive Myelopathy and Secondary Cord Ischemia: A Case Report

**DOI:** 10.1155/cris/5863091

**Published:** 2026-06-08

**Authors:** Molly E. Milano, Alec M. Giakas, Eric Tecce, Jonathan Dalton, I. David Kaye

**Affiliations:** ^1^ Thomas Jefferson University Hospital, 111 S. 11th Street, Philadelphia, 19107, Pennsylvania, USA, jefferson.edu; ^2^ Rothman Orthopaedic Institute, 925 Chestnut Street, 5th Floor, Philadelphia, 19107, Pennsylvania, USA, rothmanortho.com

## Abstract

Spinal cord infarction (SCI) is an uncommon condition with a generally poor prognosis for neurologic recovery. There are numerous possible causes for SCI, yet frequently the etiology is not fully elucidated. Literature regarding SCI is sparse, with no conclusive algorithmic strategies for diagnosis and treatment. This case report describes the hospital course of a male in his seventh decade of life who presented to our emergency department with acute onset of bilateral lower extremity paralysis and intense lower back pain due to an SCI at the T4 spinal level.

## 1. Introduction

Spinal cord infarction (SCI), also referred to as a spinal cord stroke, is an uncommon and heterogeneous condition that typically presents with acute onset of anterior spinal artery syndrome, with bilateral weakness, dissociated sensory loss, and autonomic dysfunction like bladder or bowel dysfunction [1]. A recent work by Qureshi et al. [2] estimated that the pooled incidence of SCI was 3.1 per 100,000 person‐years. Due to limited availability of universally accepted diagnostic and imaging criteria, diagnosis of SCI is typically made clinically [3–7]. Although magnetic resonance imaging (MRI) can provide important information regarding the presence, spinal level, and extent of an infarct, these factors depend on the timing of the vascular insult [3, 8, 9]. The hallmark of SCI is acute onset of neurologic deficits, with 77% of patients reaching nadir within 12 h [10]. For these reasons, prompt diagnosis of SCI can be a challenge, even for experienced spine surgery providers.

The etiology of SCI has been reported in the literature as attributable to a number of different inciting factors, including trauma, hypotension during surgery, and thrombotic/embolic phenomena in the setting of a patent foramen ovale, fat emboli after orthopaedic surgery, the use of oral contraceptive pills, malignancy, fibrocartilaginous emboli, and infection‐related ischemia [11–18]. More recent literature has shown reports of SCI or spinal cord ischemia secondary to coronavirus disease of 2019 (COVID‐19) infection [19–22].

The current report presents the findings from a case of an acute SCI in a patient with a spinal abscess and bacteremia who presented with bilateral lower extremity paralysis and a loss of sensation below the T4 dermatome. The patient’s condition was addressed with timely spinal cord decompression and appropriate antibiotics, but he did not return to his baseline functional status. This report was written in accordance with ICMJE guidelines for anonymization [23].

## 2. Case Presentation

A male in his seventh decade of life with a past medical history significant for obesity, type 2 diabetes mellitus, hypertension, cirrhosis, and chronic back pain presented to the emergency department as a transfer for sudden onset of bilateral lower extremity weakness. His surgical history included prior L2‐L3 lateral lumbar interbody fusion in 2016 and a L2‐S1 posterior lumbar decompression with a L3‐L5 posterolateral lumbar fusion in 2017. Both of these procedures were at an outside institution. 3 weeks prior to admission, the patient presented to an outside outpatient facility for worsening back pain and lower extremity radiculopathy but did not seek admission at that time. On the morning of admission, he reported waking up and being unable to move his legs, which prompted the emergency department visit at our institution.

On initial examination, there was thoracic spine tenderness with no cervical or lumbar tenderness. The patient had diminished/absent sensation from the nipple line down. There was also a lack of sensation in the genital, perineum, and perianal regions. The patient had diminished reflexes in the bilateral lower extremities, and he had a loss of rectal tone. On motor examination, the patient had diffuse 0/5 strength in his bilateral lower extremities, with 5/5 strength throughout his bilateral upper extremities.

Laboratory values upon presentation demonstrated a white blood cell count of 4500 (4500–11,000 WBC/µL) with 79.8% neutrophils (40%–60%) and 5% lymphocytes (20%–40%), an ESR of 69 (0–20 mm/h), and CRP of 27.6 (<0.3 mg/dL). Our patient’s coagulation profile demonstrated a prothrombin time (PT) of 14.9 s (9.4–13 s), partial thromboplastin time (PTT) of 35 s (25–27 s), and international normalized ratio (INR) of 1.34 (0.84–1.16).

**Figure 1:**
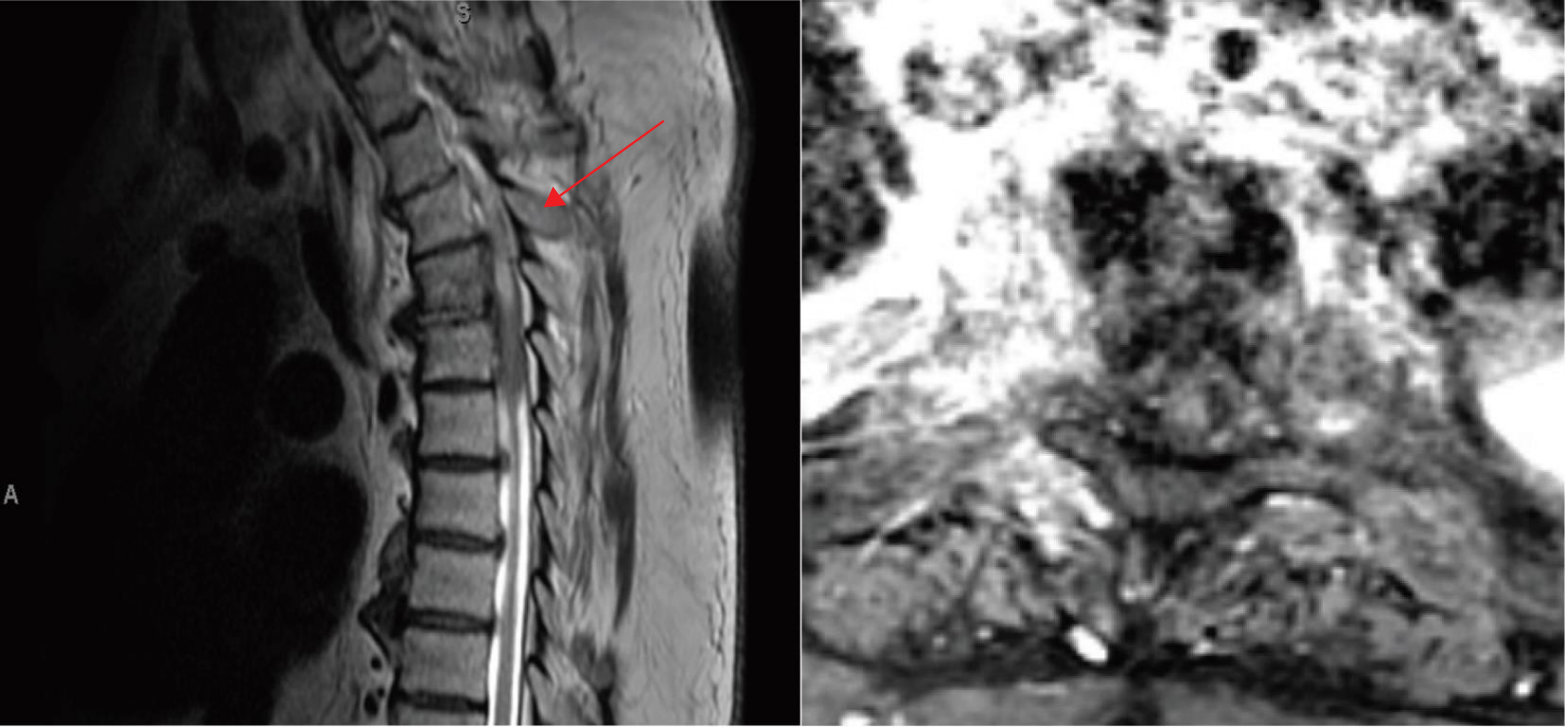
Radiological findings in patient presenting with lower extremity weakness.

MRI reports from the outside hospital described a mild hyperintensity within the disc space at T4‐T5 and possibly T3‐T4 with severe spinal canal stenosis and cord compression. Repeat MRI at our institution showed findings consistent with T4‐T5 cord compression, SCI centered at T4, and a possible epidural abscess (Figure 1). Blood cultures were positive for methicillin‐susceptible *Staphylococcus aureus* (MSSA) bacteremia.

**Figure 2:**
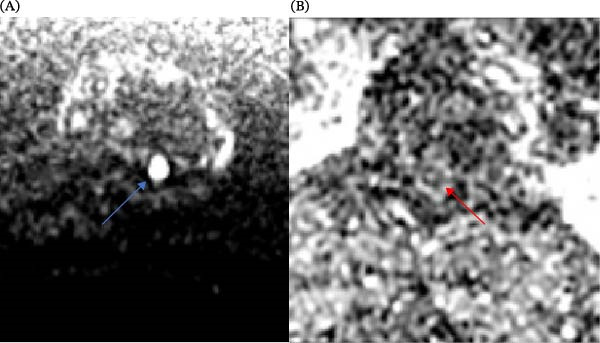
DWI (A) and ADC (B) imaging findings.

The differential diagnosis for the patient’s bilateral lower extremity paralysis was broad upon arrival to the emergency department. It included cauda equina syndrome, a compressive abscess or malignancy causing myelopathy, an epidural fluid collection/hematoma, and a SCI. The patient’s sudden bilateral lower extremity motor loss with a loss of control of bowel function, bladder function, and absent perineal sensation was initially concerning for an acute cauda equina syndrome. However, MRI of the lumbar spine demonstrated no high‐grade stenosis or compression of the neural elements, and MRI of the thoracic spine demonstrated a T2 hyperintensity within the T4‐T5 disc space with dural/epidural/subarachnoid enhancement, concerning for discitis. Additionally, there was a mass effect on the spinal cord at T4‐T5 with cord edema concerning for a SCI, as well as a “pencil‐like” hyperintensity seen on T2 imaging, a highly sensitive imaging finding seen in SCI [10]. Diffusion‐weighted imaging (DWI) and apparent diffusion coefficient (ADC) findings demonstrate a notable diffusion restriction and corresponding low ADC, respectively, consistent with findings seen in SCI (Figure 2) [10]. The final MRI read describing a SCI centered at T4 and accompanying findings on DWI and ADC images consistent with SCI, were consistent with the patient’s presentation with complete motor and sensory loss below the nipple line/T4 dermatome and myotome.

Axial and sagittal cuts of an MRI of the thoracic spine with contrast demonstrate epidural abscess with spinal cord compression and an area of T2 high signal intensity consistent with spinal cord infarct. Sagittal cuts are notable for showing “pencil‐like” hyperintensity, a sensitive finding in SCI (red arrow).

Axial DWI demonstrating notable diffusion restriction (Figure 2A—blue arrow), with corresponding low ADC values (Figure 2B—red arrow), supporting diagnosis of acute SCI.

The patient underwent a T3‐T6 central decompression with partial medial facetectomies by the orthopaedic spine surgery service within 24 h of admission.

There was an infectious‐appearing fluid collection that was encountered after removal of the lamina. The fluid was sent for culture, which was positive for MSSA. Additionally, bone and tissue were sent for pathology, which was negative for malignancy, bacterial growth, or fungal growth. During surgery, an ultrasound was used with results interpreted intraoperatively by our neurosurgery colleagues, which confirmed cord edema and adequate decompression but ruled out further evidence of other concerning fluid collections or intramedullary processes. A deep drain was placed, and the wound was closed. The patient was sent to the intensive care unit for monitoring of mean arterial pressure (MAP) goals postoperatively (MAP goal > 80 mmHg). Postoperatively the patient was placed on cefazolin that was later adjusted to nafcillin 2 g IV q4 by ID based on the positive intraoperative MSSA findings.

On hospitalization day 6 (postoperative day 4), the patient was evaluated by physical and occupational therapy. He worked with physical and occupational therapy 5–7 days a week for the remainder of his hospitalization until his discharge to a skilled nursing facility for short‐term rehabilitation.

The patient was discharged on hospitalization day 37 in stable condition to a skilled nursing facility for short‐term rehabilitation. The patient remained in acute spinal cord rehabilitation for ~1 month. The patient’s spinal incision healed well. The patient’s motor exam was unchanged—he remained 0/5 strength diffusely in his lower extremities. There was no sensation to light touch below the T4 dermatome. Additionally, he did not regain control of his bowel or bladder, and a suprapubic catheter was placed.

## 3. Discussion

SCI is an uncommon disease process that varies significantly in clinical presentation, severity, and outcome from patient to patient [24]. The current work describes the case of a patient who experienced acute onset of loss of motor function and sensation below the T4 level in the setting of MRI findings concerning for both a spinal infection and SCI. Intraoperative findings confirmed the presence of infectious‐appearing fluid during spinal decompression, which ultimately grew MSSA on cultures.

Reports of SCI connected to infectious disease processes have been scarcely detailed in the literature. A small number of articles have described SCIs in the presence of bacterial meningitis [25–28]. The authors of these studies theorized that the infarctions may have been caused by inflammatory vasculitis inducing activation of the coagulation cascade, systemic hypotension due to shock, or arachnoiditis with secondary vasculitis and arteriospasm [25–28]. Moffett and Berkowitz [27] reviewed 22 cases of spinal cord dysfunction complicating bacterial meningitis and concluded that ischemia of the cord resulting from vasculitis was the probable cause. Similarly, Van Egeraat et al. [29] described SCI as a complication of meningococcal meningitis, noting that vascular mechanisms and coagulation abnormalities play an important role in the etiology. The mechanism for infection‐associated thrombosis outside of the spine has been described in the literature, which theorizes that infectious pathogens induce a massive proinflammatory state which triggers the activation of platelets in the setting of endothelial damage, leading to the deposition of fibrin and eventual thrombus formation [30, 31].

Consequently, it is possible that the patient’s underlying epidural infection invoked an inflammatory response that spread beyond the site of abscess, leading to irritation and vasospasm of the arachnoid mater. Alternatively, this patient’s SCI may have been secondary to the presence of a hypercoagulable state and subsequent venous congestion induced from the large surrounding infectious collection. Although our patient’s coagulation profile was not indicative of a procoagulative state, these values may not accurately capture his true local thrombotic potential. In patients with cirrhosis, conventional coagulation tests only assess procoagulant pathways, while failing to account for concurrent reductions in natural anticoagulants (protein C, protein S, and antithrombin), rendering them inadequate measures of his hemostatic state [32–34]. Thus, his coagulation profile on admission cannot exclude a hypercoagulable state, which may have contributed to his SCI in the setting of his active epidural infection. The present patient had bacteremia from his epidural abscess, which likely contributed to a systemic infection and proinflammatory state, as evidenced by starkly elevated inflammatory markers, predisposing him to thrombosis at a rate of 2–20 times greater than patients without infection [30, 31]. Iwasaki et al. [35] present a case of an SCI caused by an epidural abscess with resultant spinal artery occlusion, confirmed on angiography, likely from a bacterial embolism. SCIs as a result of COVID‐19 have recently been discussed in the literature. The authors of these reports state that the respective SCIs were caused by coagulopathy as a result of COVID‐19 infection in the setting of elevated fibrinogen and D‐dimer levels [21, 22]. Similar to COVID‐19, our patient’s spinal stroke may have been caused by an infection resulting in an inflammation‐induced hypercoagulable state but may be more exactly explained by a localized hypercoagulable state secondary to localized spine infection‐related endothelial damage.

Localized infection has been well described in the literature as causing vascular endothelial damage and thrombosis through several mechanisms. In the case of a spinal epidural abscess specifically, vasculitic infarction and septic thrombophlebitis have been recognized as pathways to spinal cord injury beyond mechanical compression alone [36, 37]. Bacterial pathogens trigger a cascade of endothelial injury that transforms the local vascular endothelium from an anticoagulant to procoagulant surface through degradation of the protective endothelial glycocalyx, upregulation of tissue factor expression, and loss of natural anticoagulants like thrombomodulin and the endothelial protein C receptor [38, 39]. Additionally, recruited neutrophils can release neutrophil extracellular traps that act as a scaffold for platelet aggregation and thrombin activation, producing microthrombi that can occlude microvasculature supplying the spinal cord [40]. In the case of our patient, where adequate surgical decompression and appropriate antibiotic therapy failed to produce neurologic recovery, irreversible ischemic injury from infection‐induced thrombosis represents the most plausible explanation for his persistent deficits.

The patient had a documented clinic visit for ongoing bilateral lower extremity radiculopathy just 3 weeks prior to presentation. Given the time course, it is possible that these new symptoms were related to an ongoing spinal infectious process/abscess causing progressive compressive myelopathy. However, his acute onset of worsening of symptoms on presentation to the emergency department was likely due to a more acute process, like infarction. We hypothesize that our patient’s SCI was likely a secondary phenomenon because of both spinal infection and subsequent progressive compression caused by the patient’s epidural abscess. Nonetheless, given the ongoing infection and stenosis, the decision was made to decompress the spinal cord. Unfortunately, as in most SCI cases that present with paralysis, the patient did not experience substantial neurologic recovery. Our patient’s lower extremity pain and weakness experienced weeks before presentation could have been due to compressive myelopathy or cord edema as a result of the ongoing infectious process. However, despite adequate decompression and treatment of infection, he did not achieve meaningful neurologic improvement. His persistent deficits are likely attributable to a SCI in the setting of an epidural abscess and local endothelial damage promoting thrombosis. Furthermore, his advanced age and underlying diabetes mellitus, a well‐established risk factor for microvascular disease and impaired neurologic recovery, likely contributed to his poor functional outcomes. These comorbidities may have increased his susceptibility to ischemic injury by compromising collateral spinal cord perfusion and impairing the capacity for neuronal repair following the severe compressive insult.

Regarding imaging findings, our patient demonstrated a pencil‐like T2‐hyperintensity on sagittal MRI, a pattern highly sensitive for SCI, present in up to 98%–100% of anterior spinal artery territory infarcts [41, 42]. Additionally, the DWI images revealed a notable hyperintensity with corresponding low values on ADC mapping, findings indicative of true restricted diffusion consistent with acute ischemia [43, 44]. This DWI/ADC pattern is especially important for distinguishing SCI from inflammatory myelopathies. In infarction, DWI hyperintensities demonstrate corresponding ADC hypointensities, whereas inflammatory lesions typically show isointense or elevated ADC values [44]. While the classic “owl eye” sign on axial imaging is often cited as characteristic of SCI, and is not clearly seen in our patient’s imaging, it is present in only 37%–68% of cases and is neither specific nor a prerequisite for diagnosis [10, 42]. The absence of a definitive owl eye pattern in our patient does not exclude SCI, particularly given the presence of pencil‐like hyperintensity, true diffusion restriction with low ADC values, clinical presentation consistent with anterior spinal artery involvement, and persistent neurologic deficits despite adequate surgical decompression and antibiotic therapy [10].

Amongst the small amount of literature describing outcomes after SCI, the prognosis is generally considered poor [1]. A study by Robertson et al. [1] examined 115 patients with SCI between 1990 and 2007 who initially presented with a wide range of American Spinal Injury Association (ASIA) scores. At final follow‐up, 23% of patients had died—among survivors, 42% were using a wheelchair and over half needed a bladder catheter [1]. Amongst patients who required a wheelchair upon discharge from their initial admission, only 5% were able to walk unaided at final follow‐up [1].

Emerging therapies such as stem cells, biomaterials, and neuroprotective drugs continue to be explored in the setting of spinal cord injury [45]. Stem cells in particular have shown promise due to their neuroregenerative and neuroprotective effects in preclinical work. Unfortunately, these treatments still suffer from poor cell survival and difficulty with integration into the surrounding spinal cord parenchyma [46]. Additionally, stem cell lineages have substantial genetic heterogeneity, which can impact their clinical efficacy [47, 48]. Despite these challenges, continued research is critically important in order to identify therapies for spinal cord injuries.

## Funding

No specific funding was received for the writing of this case report.

## Consent

This report was written in accordance with ICMJE guidelines for anonymization and, thus, does not require direct patient consent.

## Conflicts of Interest

The authors declare no conflicts of interest.

## Data Availability

The data that support the findings of this study are available from the corresponding author upon reasonable request.
